# Concentration-Response Relationships of Dolutegravir and Efavirenz with Weight Change After Starting Antiretroviral Therapy

**DOI:** 10.1111/bcp.15177

**Published:** 2022-01-26

**Authors:** Rulan Griesel, Aida N Kawuma, Roeland Wasmann, Simiso Sokhela, Godspower Akpomiemie, WD Francois Venter, Lubbe Wiesner, Paolo Denti, Phumla Sinxadi, Gary Maartens

**Affiliations:** 1 Division of Clinical Pharmacology, Department of Medicine, University of Cape Town, Cape Town, South Africa; 2 Wellcome Centre for Infectious Diseases Research in Africa, Institute of Infectious Disease and Molecular Medicine, University of Cape Town, Cape Town, South Africa; 3 Ezintsha, Wits Reproductive Health and HIV Institute, Faculty of Health Sciences, University of the Witwatersrand, Johannesburg, South Africa

**Keywords:** dolutegravir, efavirenz, weight gain, concentration-response relationship, visceral adipose tissue, subcutaneous adipose tissue

## Abstract

**Aim:**

Dolutegravir is associated with more weight gain than efavirenz in people starting antiretroviral therapy (ART). We investigated the concentration-response relationships of efavirenz and dolutegravir with weight gain.

**Methods:**

We determined concentration-response relationships of dolutegravir and efavirenz (both combined with tenofovir disoproxil fumarate and emtricitabine) with changes in weight and fat distribution, derived from dual-energy x-ray absorptiometry scans, in a nested study of ART-naïve participants from a randomised controlled trial. Pharmacokinetic parameters used in analyses were efavirenz mid-dosing interval (MDI) concentrations and estimated dolutegravir area under the concentration-time curve (AUC_0-24_) using a population pharmacokinetic model developed in the study population. Study outcomes were percentage changes from baseline to week 48 in weight, and visceral and subcutaneous adipose tissue (VAT and SAT) mass.

**Results:**

Pharmacokinetic data were available for 158 and 233 participants in the efavirenz arm and dolutegravir arms respectively; 57.0% were women. On multivariable linear regression there were independent negative associations between efavirenz concentrations and changes in both weight (P <0.001) and SAT mass (P = 0.002). Estimated dolutegravir AUC_0-24_ was not associated with change in weight (P = 0.109) but was negatively associated with change in VAT mass (P = 0.025).

**Conclusion:**

We found an independent negative concentration-response relationship between efavirenz concentrations and weight change in ART-naïve participants. Dolutegravir concentrations were not independently associated with weight change. These findings suggest that weight gain differences between efavirenz and dolutegravir are driven by efavirenz toxicity impairing weight gain rather than by off-target effects of dolutegravir causing weight gain.

## Introduction

Integrase strand transfer inhibitors (InSTIs) are associated with more weight gain than other classes of antiretrovirals among people living with HIV (PLWH) initiating antiretroviral therapy (ART) in randomised controlled trials.(1–3) Two randomised controlled trials conducted in ART-naïve PLWH in sub-Saharan Africa reported that dolutegravir-based regimens were associated with more weight gain (especially among women) than efavirenz-based regimens.(2,3) There are two potential explanations for the greater weight gain with dolutegravir than efavirenz: dolutegravir may have off-target effects that stimulate appetite or perturb metabolism, or efavirenz may impair weight gain through its metabolic or neuropsychiatric toxic effects. Dolutegravir inhibits the melanocortin 4 receptor (MC4R),(4) which is associated with energy homeostasis and appetite regulation.(5,6) Efavirenz causes concentration-dependent mitochondrial toxicity, impaired adipocyte differentiation, and neuropsychiatric adverse drug reactions that could impair appetite.(7–9)

Efavirenz is primarily metabolised by the cytochrome P450 2B6 enzyme (CYP2B6). Polymorphisms coding loss-of-function in the *CYP2B6* gene result in higher efavirenz concentrations.(10) Leonard *et al*. reported greater weight gain among *CYP2B6* slow metabolisers on efavirenz-based ART when switched to an InSTI-based regimen.(11) We recently showed that *CYP2B6* metaboliser genotype was strongly associated with weight change among PLWH starting efavirenz-based ART: extensive metabolisers gained the most weight, and slow metabolisers lost weight.(12) We also observed that *CYP2B6* extensive metabolisers in the efavirenz arm had similar weight gain to participants in the dolutegravir arm, supporting the hypothesis that weight gain on dolutegravir-based ART is not due to off target effects of dolutegravir, but rather that impaired weight gain on efavirenz-based ART is due to concentration-dependent efavirenz toxicity. These two studies showing associations between *CYP2B6* metaboliser genotype and weight gain differences between InSTIs and efavirenz suggest, but do not confirm, an inverse concentration-response relationship between efavirenz and weight gain. Establishing a concentration-response relationship is important as it is one of Bradford Hill’s criteria for establishing causation.(13)

We hypothesised that among PLWH initiating ART there is an inverse concentration-response relationship between efavirenz and weight gain, and that there is no concentration-response relationship between dolutegravir and weight gain. We determined efavirenz and dolutegravir drug concentrations in participants from the ADVANCE study(3) on identical nucleoside reverse transcriptase inhibitors (emtricitabine and tenofovir disoproxil fumarate [TDF]) to determine concentration-response relationships with change in weight over 48 weeks.

## Methods

### Study Design and Participants

ADVANCE was an open-label randomised controlled trial conducted in Johannesburg, South Africa.(3) ART-naïve participants were randomised to one of three arms: 1) dolutegravir, TDF, and emtricitabine; 2) dolutegravir, tenofovir alafenamide (TAF), and emtricitabine; and 3) efavirenz, TDF, and emtricitabine. Trial inclusion criteria were: age ≥12 years, no ART use in the previous 6 months, a creatinine clearance of >60 mL/minute, and human immunodeficiency virus type 1 (HIV-1) RNA ≥500 copies/mL.

Inclusion criteria for this sub-study were: adults (age ≥18 years); participants from the dolutegravir, TDF, and emtricitabine arm who had sparse dolutegravir plasma samples; participants from the efavirenz arm who consented to genomic testing and had efavirenz mid-dosing interval plasma samples available; baseline and week 48 anthropometric and dual energy X-ray absorptiometry (DXA) scan data. Exclusion criteria for this sub-study were: participants with dolutegravir or efavirenz concentrations below the lower limit of quantification (LLOQ) of the assays; women who became pregnant during the first 48 weeks of follow-up; and participants who received rifampicin-based antituberculosis therapy during the first 48 weeks of follow-up.

### Drug Concentration Analyses

Dolutegravir plasma concentrations were determined by a validated liquid chromatography tandem mass spectrometry assay. Samples were processed with a liquid-liquid extraction method using dolutegravir-d4 as internal standard, followed by high performance liquid chromatography with tandem mass spectrometry detection using an AB SCIEX API 4000 instrument. The analyte and internal standard were monitored at mass transitions of the protonated precursor ions m/z 420.1 and m/z 424.2 to the product ions m/z 277.2 and m/z 279.1, respectively. The calibration curve fitted a quadratic regression over the range 0.03-10.0 μg/mL. The combined accuracy (%Nom) and precision (%CV) statistics of the quality control samples during validation were between 103.5% and 106.0%, and 4.6% and 6.1%, respectively. Efavirenz plasma concentrations were determined by a validated liquid chromatography tandem mass spectrometry method as described by Bienczak *et al*.(14) All assays were done at the Division of Clinical Pharmacology, University of Cape Town - the laboratory participated in the Clinical Pharmacology Quality Assurance (CPQA) external quality control program under a contract with the Division of AIDS of the National Institute of Allergy and Infectious Diseases. Both assays were CPQA approved.

### Pharmacokinetic Determinants and Modelling

Forty-one participants from both dolutegravir arms (21:20) were enrolled in an intensively sampled pharmacokinetic sub-study nested within ADVANCE (samples drawn at pre-dose, 1, 2, 4, 6, 8, and 24 hours post-dose). Two-hundred-and-sixteen other patients underwent sparse sampling at weeks 24 and 48, with participant’s self-reporting time of their last dolutegravir dose. The intensively sampled data were used to develop a population pharmacokinetic model of dolutegravir, which was then applied to all available pharmacokinetic data (including the sparse samples) to produce individual estimates of steady-state area under the concentration-time curve over 24 hours (AUC_0-24_). For more details about the modelling and the procedure to obtain the individual exposure, please consult the supplementary material (Supplemental Table 1, Supplemental Figures 1 and 2). In the efavirenz arm, efavirenz mid-dosing interval plasma concentrations (approximately 12 hours after self-reported time of last efavirenz dose) were taken at week 24 or 48.

### Study Outcomes and Definitions

We calculated percentage change in weight from baseline to week 48. Body composition measures using DXA (Discovery DXA System^®^, software version APEX 4.6.0.1, Hologic, Bedford, MA, USA) at baseline and week 48 were used to estimate changes in abdominal visceral adipose tissue (VAT) and subcutaneous adipose tissue (SAT).(12,15) The percentage change in mass from baseline to week 48 was calculated for VAT and SAT. Participants in the efavirenz arm were categorised by three genetic loss-of-function polymorphisms in *CYP2B6* as extensive, intermediate, and slow metabolisers.(12)

### Statistical Analysis

All statistical analyses were performed using Stata (version 16.0; StataCorp: Stata Statistical Software, College Station, TX, USA). Graphs were made using GraphPad Prism (version 9.0; GraphPad Software, San Diego, CA, USA). Medians with interquartile ranges (IQR) were used to describe all continuous variables. Proportions were used to describe categorical data. Outcome variables included: percentage change in weight from baseline to week 48, as well as percentage change in VAT and SAT from baseline to week 48. We utilised the 2-sample Wilcoxon rank-sum test to compare outcome variables between participants in the dolutegravir and efavirenz arms. We used two-way scatter plots and Spearman’s rank-order correlation (*r_s_*) to visually assess efavirenz mid-dosing interval plasma concentrations and dolutegravir AUC_0-24_ estimates with percentage change in weight form baseline to week 48.

Univariable linear regression models with robust standard errors were used to assess associations between log transformed efavirenz mid-dosing interval plasma concentrations and dolutegravir AUC_0-24_ estimates and percentage change from baseline to week 48 (weight, SAT and VAT) in the efavirenz and dolutegravir arms, respectively. In multivariable regression models, we adjusted for the following covariates that were selected *a priori*: age; sex; baseline body mass index (BMI), CD4 count, and HIV-1 RNA.

## Results

The ADVANCE study enrolled 351 participants into the efavirenz, emtricitabine and TDF arm, and 351 participants into the dolutegravir, emtricitabine and TDF arm. One-hundred-and-seventy participants from the efavirenz arm had efavirenz mid-dosing interval plasma concentrations. Two-hundred-and-thirty-six participants from the dolutegravir arm had AUC_0-24_ estimates available (20 of these were part of the intensely sampled pharmacokinetic sub-study). Sixteen participants from the efavirenz arm and five from the dolutegravir arm were excluded from analyses (Supplemental Figure 3). A further seven participants from the efavirenz arm and six from the dolutegravir arm did not have baseline or week 48 DXA scan results available. The baseline characteristics and percentage weight gain from baseline to week 48 of enrolled participants did not significantly differ from those not enrolled (Supplemental Table 2 and 3). Baseline characteristics of the included participants are shown in Table 1.

### Pharmacokinetic Assessment

All included participants in the efavirenz arm took their dose the evening before sampling and had detectable efavirenz plasma concentrations. The median time from dose to sampling was 13.9 hours (IQR 12.8 to 15.2) for those with available data (146/154). The median efavirenz mid-dosing interval plasma concentration from the available samples was 2.7 μg/mL (IQR 1.8 to 5.6). The median efavirenz mid-dosing interval plasma concentrations stratified by *CYP2B6* metaboliser genotype followed an expected distribution: extensive metabolisers 1.8 μg/mL (IQR 1.4 to 2.3); intermediate metabolisers 2.6 μg/mL (IQR 1.9 to 3.8); slow metabolisers 8.0 μg/mL (IQR 5.6 to 13.4). The median dolutegravir AUC_0-24_ estimate from the available samples was 67.2 mg·h/L (IQR 54.0 to 95.3).

### Weight Assessment

**Figure 1:**
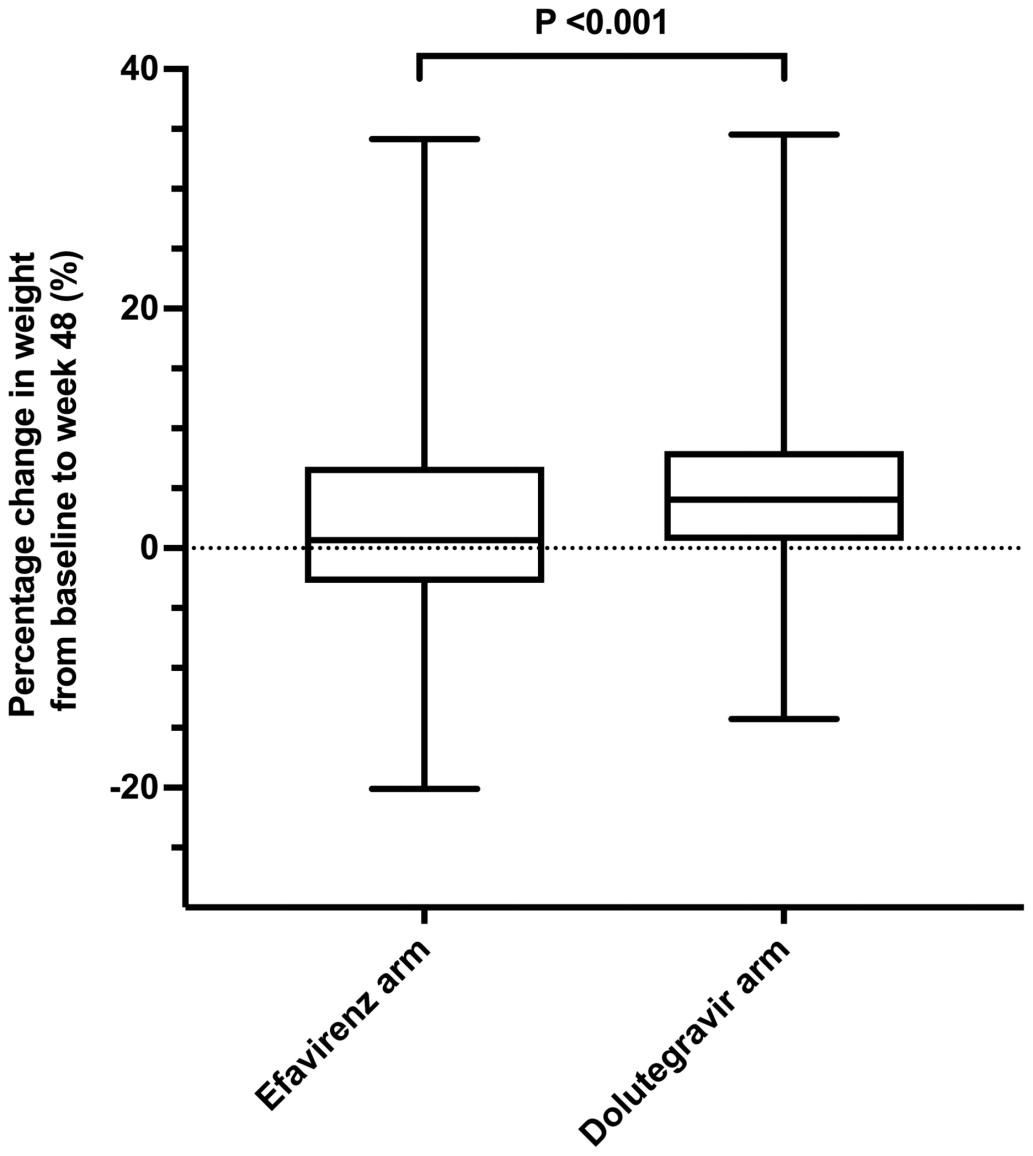
Percentage change in weight from baseline to week 48 among participants in the efavirenz arm (n=154) and in the dolutegravir arm (n=231).

Participants in the dolutegravir arm gained more weight than those in the efavirenz arm (percentage change in median weight from baseline to week 48: 4.0% (IQR 0.6 to 8.1) versus 0.7% (IQR -2.9 to 6.8), respectively (Wilcoxon rank-sum P <0.001) (Figure 1).

**Figure 2:**
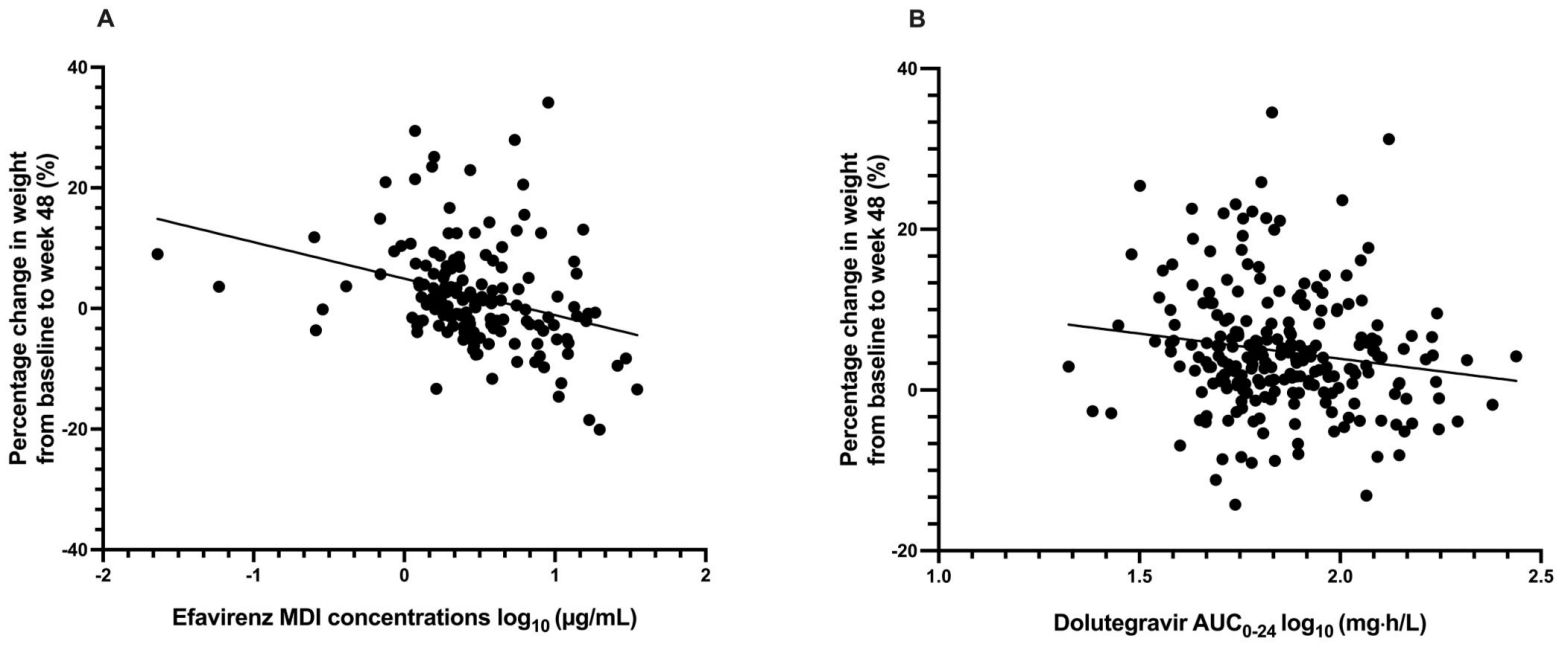
Scatter plot of percentage change in weight from baseline to week 48 by drug exposure among participants in the efavirenz arm (n=154) (*r_s_* = -0.377 [95% CI -0.509 to -0.228], P <0.001) (panel A) and among participants in the dolutegravir arm (n=231) (*r_s_* = -0.159 [95% CI -0.286 to - 0.027], P = 0.016) (panel B). The straight line represents the univariable linear regression line. (MDI = mid-dosing interval plasma concentrations, AUC_0-24_ = area under the concentration-time curve)

Higher efavirenz mid-dosing intervaI plasma concentrations and dolutegravir AUC_0-24_ were correlated with a decrease in percentage change in weight from baseline to week 48 for the efavirenz (*r_s_* = -0.377 [95% CI -0.509 to -0.228], P <0.001) and dolutegravir (*r*_s_ = -0.159 [95% CI -0.286 to -0.027], P = 0.016) arms, respectively (Figure 2). On multivariable linear regression (adjusting for age, sex, baseline BMI and CD4 count and HIV-1 RNA) the negative association between efavirenz mid-dosing interval plasma concentrations and percentage change in weight from baseline to week 48 remained significant (Table 2).

Increasing age was an independent predictor of weight gain on multivariable linear regression in the dolutegravir arm but age was not significantly associated with weight change in the efavirenz arm (Table 2). Univariable linear regression in the efavirenz arm showed that increasing age was associated with weight loss among *CYP2B6* slow metabolisers (n=41) (estimate = -2.635 [95% CI -5.093 to -0.177], P = 0.036), but age was not associated with weight change among extensive and intermediate metabolisers combined (n=113) (estimate = 0.023 [95% CI -1.029 to 1.075], P = 0.966), or separately: extensive metabolisers (n=46) (estimate = -0.278 [95% CI -2.508 to 1.952], P = 0.803) and intermediate metabolisers (n=67) (estimate = 3.449 [95% CI -4.264 to 11.162], P = 0.375). Baseline CD4 count and HIV-1 RNA were independently associated with weight change from baseline to week 48 in both the efavirenz and the dolutegravir arms (Table 2).

### Fat Distribution Assesment

**Figure 3:**
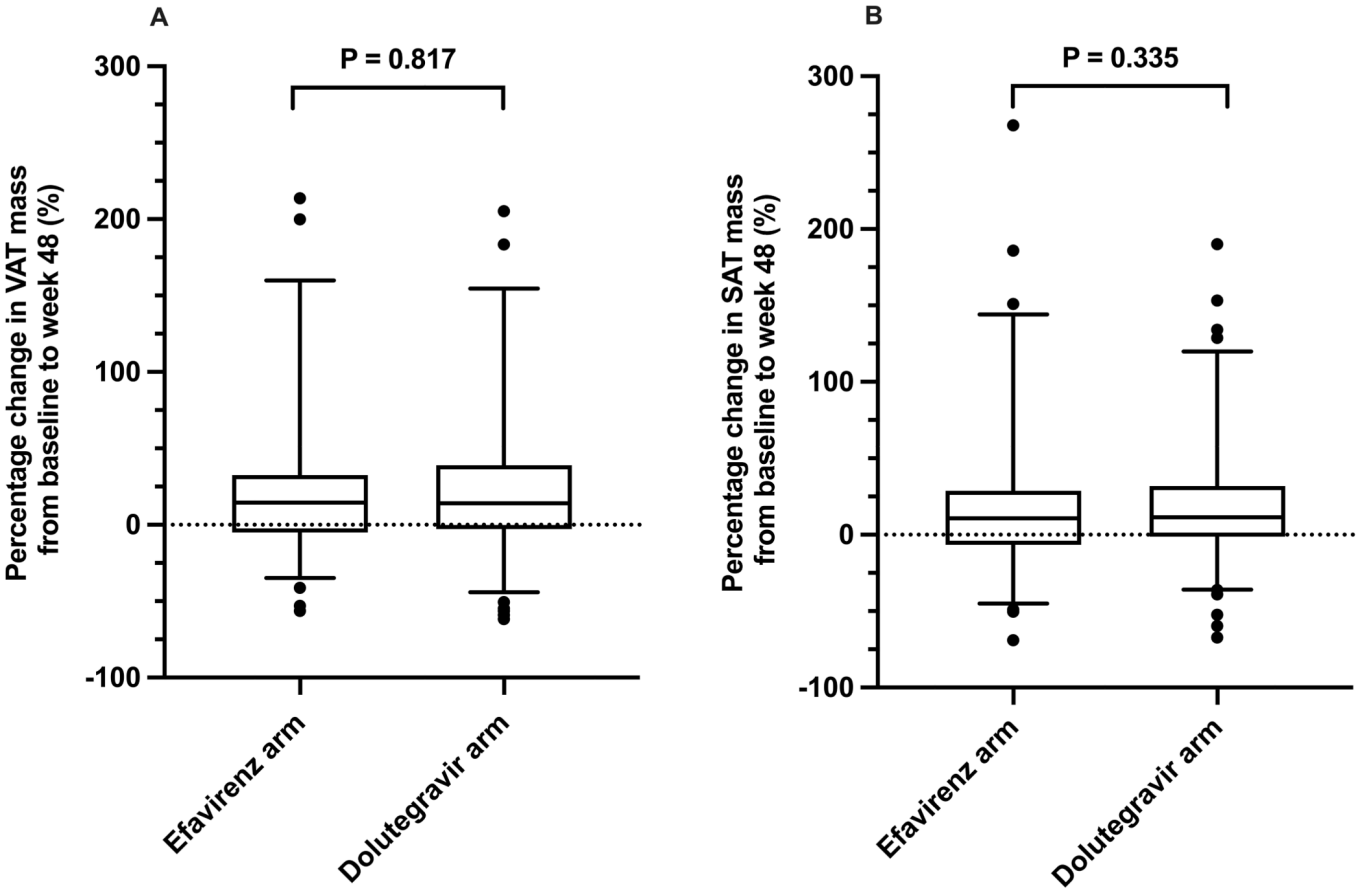
Percentage change in VAT mass (panel A) and SAT mass (panel B) from baseline to week 48 among participants in the efavirenz arm (n=152) and participants in the dolutegravir arm (n=227) (VAT = visceral adipose tissue, SAT = subcutaneous adipose tissue).

In the efavirenz arm the median change from baseline to week 48 in percentage VAT mass was 14.5% (IQR -5.0 to 32.3) (Figure 3A) and in percentage SAT mass was 10.8% (IQR -6.3 to 28.6) (Figure 3B). In the dolutegravir arm the median change from baseline to week 48 in percentage VAT mass was 14.2% (IQR -2.7 to 39.0) (Figure 3A) and in percentage SAT mass was 11.5% (IQR -1.3 to 31.8) (Figure 3B).

Univariable and multivariable linear regression analyses of associations with VAT and SAT are shown in Tables 3 and 4 respectively. There was an independent negative association between percentage change in SAT mass and efavirenz mid-dosing interval plasma concentrations on multivariable linear regression. Baseline BMI was negatively associated with percentage change in SAT mass in both arms on multivariable linear regression. Baseline HIV-1 RNA was positively associated with percentage change in SAT mass in the dolutegravir arm on multivariable linear regression. There was an independent negative association between percentage change in VAT mass and dolutegravir AUC_0-24_ estimates on multivariable linear regression; however, this association was no longer statistically significant in a sensitivity analysis excluding three outlier participants with an increase of >300% in VAT mass from baseline to week 48. Baseline BMI was negatively associated with percentage change in VAT mass in both arms on multivariable linear regression.

## Discusssion

We found independent negative associations between efavirenz concentrations and change in weight and SAT mass after starting ART. Dolutegravir AUC_0-24_ was negatively correlated with change in weight on univariable analysis; however, this association did not remain significant on multivariable analysis. These findings suggest that weight gain differences between efavirenz and dolutegravir are driven by efavirenz toxicity impairing weight gain rather than by off-target effects of dolutegravir causing weight gain.

Our finding that increasing efavirenz concentrations were negatively associated with weight change is similar to those of a recent Taiwanese study of virologically suppressed participants on efavirenz-based ART over 192 weeks.(16) Interestingly, the Taiwanese study failed to find an association between the *CYP2B6* 516G→T genotype and weight change, likely due to a low prevalence of the GT and TT polymorphism in the cohort (18.6% and 0%, respectively) and the lack of a more detailed *CYP2B6* genotypic analysis. Significant associations between weight change and *CYP2B6* metaboliser genotype, which is a proxy for efavirenz exposure, have been shown among PLWH switched from an efavirenz- to an InSTI-based ART regimen(11), and among ART-naïve participants in the ADVANCE study initiating an efavirenz-based regimen.(12) Impaired weight gain with high efavirenz exposure could be explained by the adipocyte mitochondrial dysfunction, decreased adiponectin expression, and increased proinflammatory cytokine release.(17) Lipoatrophy has been associated more frequently with efavirenz-based than protease inhibitor-based ART regimens.(18,19) The ADVANCE study results reported that participants in the efavirenz arm gained less limb fat up to week 96 than those in the two dolutegravir arms.(20) Another potential reason for poor weight gain with higher efavirenz concentrations is the drug’s known neuropsychiatric side effect profile;(9) however the week 96 ADVANCE study findings reported that appetite, nausea, and insomnia were not significantly associated with weight change.(20)

InSTIs have been associated with more weight gain than protease inhibitor- or non-nucleoside reverse transcriptase inhibitor-based regimens among treatment-naïve participants initiating ART(1) and among treatment-experienced participants switched to an InSTI-based regimen.(21) Weight gain with dolutegravir has been attributed to off-target inhibition of the endogenous ligand binding to MC4R. However, concentrations of dolutegravir required to inhibit MC4R are well above predicted clinical exposure, making this mechanism implausible.(22) Other mechanisms which have been proposed include: a return to health effect where weight gain is associated with clinical recovery (supported by the findings of greater weight gain among participants with lower baseline CD4 counts and higher baseline HIV-1 RNA values), improved tolerability of newer antiretrovirals, gut microbiome disturbances and immunologic changes, and effects on adipogenesis.(21)

We found an independent association between increasing age and weight gain in the dolutegravir arm, but not in the efavirenz arm. In the efavirenz arm, increasing age was associated with weight loss among *CYP2B6* slow metabolisers, but weight change was not associated with increasing age among extensive and intermediate metabolisers.

Our finding of a negative correlation with increasing dolutegravir AUC_0-24_ and weight change was unexpected. However, this association was not significant on multivariable analysis. We also found an independent negative association between estimated dolutegravir AUC_0-24_ and change in VAT mass. However, when performing a sensitivity analysis excluding outliers, this association was no longer statistically significantly. A study of switching virologically suppressed patients to lamivudine and dolutegravir (ANRS 167 Lamidol Trial) found no significant association between trough dolutegravir or lamivudine plasma concentrations and weight gain.(23)

Our study has limitations. First, this was a post-hoc analysis, and we did not do a formal sample size estimation. Second, we did not have pharmacokinetic data available from all participants in the two included ADVANCE study arms; however, participants were enrolled into the pharmacokinetic sub-study if they consented to genetic testing and were not selected by baseline characteristics. Third, participants were on isoniazid preventive therapy for 48 weeks, which causes a drug-drug interaction with efavirenz in *CYP2B6* slow metabolisers, resulting in a ~50% increase in efavirenz concentrations.(24,25) Fourth, dolutegravir sparse samples were taken either at week 24 or week 48 to estimate AUC_0-24_; it is possible that the increase in weight from week 24 to week 48 could have had an influence on dolutegravir’s exposure secondary to increased volume of distribution. However, we feel that this is unlikely as the population pharmacokinetic model used to estimate dolutegravir AUC_0-24_ was based on individual participants’ fat free mass at the time of sampling. Finally, all our participants were African and there was a high proportion of women; our findings may not be generalisable to other populations.

In conclusion, we found an independent concentration-response relationship between efavirenz and changes in weight over 48 weeks among ART-naïve participants; higher efavirenz concentrations resulted in less gain or loss of weight over 48 weeks. The independent negative association between efavirenz concentrations and change in SAT mass suggests that adipocyte toxicity could be a mechanism for impaired weight gain. Dolutegravir exposure was not independently associated with weight change. These findings suggest that the weight gain differences between efavirenz and dolutegravir are driven by impaired weight gain due to efavirenz toxicity rather than by off-target effects of dolutegravir causing weight gain: dolutegravir allows a better return to health phenomenon.

## Supplementary Material

Supplemental material

## Figures and Tables

**Table 1:** Baseline characteristics of participants enrolled into the pharmacokinetic sub-study.

Baseline characteristics	Efavirenz arm (n=158)	Dolutegravir arm (n=233)
**Age**, years (median, IQR)	32 (28 – 37)	32 (27 – 37)
**Sex**		
Female, %	56.9	57.1
Male, %	42.1	42.9
**Black race**, %	100	100
**Weight**, kg (median, IQR)	66.8 (59.6 – 79.7)	66.0 (59.0 – 77.1)
**BMI**, kg/m^2^ (median, IQR)	23.8 (20.4 – 27.5)	22.9 (20.4 – 27.7)
**CD4 count**, cells/μL (median, IQR)	287 (169 – 403)	274 (163 – 413)
**HIV-1 RNA log_10_**, copies/mL (median, IQR)	4.5 (3.7 – 5.0)	4.4 (3.8 – 4.9)

IQR=interquartile range, BMI = body mass index, HIV = human immunodeficiency virus, RNA= ribonucleic acid

**Table 2:** Univariable and multivariable linear regression for percentage change in weight from baseline to week 48 among participants in the efavirenz arm with available efavirenz mid-dosing interval plasma concentrations (n=154) and participants with in the dolutegravir arm with available estimated dolutegravir AUC_0-24_ concentrations (n=231).

	Efavirenz arm				Dolutegravir arm			
	Univariable associations		Multivariable associations		Univariable associations		Multivariable associations	
Variable(s)	Estimate (95% CI)	p-value	Estimate (95% CI)	p-value	Estimate (95% CI)	p-value	Estimate (95% CI)	p-value
Age (per 5 years increase)	-0.684 (-1.647 to 0.278)	0.162	-0.376 (-1.297 to 0.544)	0.421	0.802 (0.237 to 1.368)	**0.006**	0.722 (0.179 to 1.264)	**0.009**
Sex								
Female	Referent group				Referent group			
Male	-1.149 (-3.866 to 1.569)	0.405	-2.804 (-5.787 to 0.179)	0.065	-0.194 (-2.212 to 1.824)	0.850	-1.080 (-3.187 to 1.027)	0.314
Baseline BMI (per 1 kg/m^2^ increase)	-0.206 (-0.417 to 0.005)	0.056	-0.224 (-0.450 to 0.001)	0.051	-0.041 (-0.196 to 0.114)	0.604	0.041 (-0.142 to 0.225)	0.656
Baseline CD4 count (per 50 cells/μL increase)	-0.558 (-0.876 to -0.239)	**0.001**	-0.429 (-0.752 to -0.106)	**0.010**	-0.395 (-0.591 to -0.199)	**<0.001**	-0.210 (-0.395 to -0.026)	**0.026**
Baseline HIV-1 RNA (per 1 log_10_ increase)	2.837 (1.281 to 4.394)	**<0.001**	2.203 (0.730 to 3.767)	**0.004**	2.851 (1.501 to 4.201)	**<0.001**	2.332 (0.916 to 3.749)	**0.001**
EFV MDI (μg/mL) (per 1 log_10_ increase)	-6.050 (-9.186 to -2.914)	**<0.001**	-6.328 (-9.672 to -2.984)	**<0.001**	–	–	–	–
DTG AUC_0-24_ (mg·h/L) (per 1 log_10_ increase)	–	–	–	–	-6.267 (-11.306 to -1.229)	**0.015**	-3.997 (-8.891 to 0.897)	0.109

AUC_0-24_ = area under the concentration-time curve, HIV = human immunodeficiency virus, RNA= ribonucleic acid, BMI = body mass index, EFV = efavirenz, MDI = mid-dosing interval concentration, DTG = dolutegravir

**Table 3:** Univariable and multivariable linear regression for percentage change in visceral adipose tissue from baseline to week 48 among participants in the efavirenz arm (n=152) and participants in the dolutegravir arm (n=227).

	Efavirenz arm				Dolutegravir arm			
	Univariable associations		Multivariable associations		Univariable associations		Multivariable associations	
Variable(s)	Estimate (95% CI)	p-value	Estimate (95% CI)	p-value	Estimate (95% CI)	p-value	Estimate (95% CI)	p-value
Age (per 5 years increase)	-2.559 (-7.264 to 2.147)	0.284	-1.575 (-6.877 to 3.726)	0.558	4.722 (0.644 to 8.801)	**0.023**	5.307 (1.373 to 9.242)	**0.008**
Sex								
Female	Referent group				Referent group			
Male	-1.176 (-17.026 to 14.675)	0.884	-10.092 (-27.827 to 7.642)	0.263	-3.645 (-18.114 to 10.825)	0.620	-13.353 (-28.863 to 2.157)	0.091
Baseline BMI (per 1 kg/m^2^ increase)	-1.633 (-2.787 to -0.480)	**0.006**	-1.772 (-2.996 to -0.548)	**0.005**	-1.224 (-2.359 to -0.090)	0.035	-2.021 (-3.566 to -0.477)	**0.011**
Baseline CD4 count (per 50 cells/μL increase)	-1.668 (-3.137 to -0.200)	**0.026**	-1.385 (-3.047 to 0.278)	0.102	-0.433 (-1.562 to 0.696)	0.450	0.650 (-0.730 to 2.029)	0.355
Baseline HIV-1 RNA (per 1 log_10_ increase)	4.940 (-5.151 to 15.029)	0.335	2.425 (-8.864 to 13.714)	0.672	4.039 (-6.020 to 14.097)	0.430	2.399 (-8.198 to 12.996)	0.656
EFV MDI (μg/mL) (per 1 log_10_ increase)	-11.542 (-24.801 to 1.717)	0.087	-12.640 (-26.527 to 1.248)	0.074	–	–	–	–
DTG AUC_0-24_ (mg·h/L) (per 1 log_10_ increase)	–	–	–	–	-38.924 (-75.533 to -2.315)	**0.037**	-42.031 (-78.682 to -5.380)	**0.025**

AUC_0-24_ = area under the concentration-time curve, HIV = human immunodeficiency virus, RNA= ribonucleic acid, BMI = body mass index, EFV = efavirenz, MDI = mid-dosing interval plasma concentration, DTG = dolutegravir

**Table 4:** Univariable and multivariable linear regression for percentage change in subcutaneous adipose tissue from baseline to week 48 among participants in the efavirenz arm (n=152) and participants in the dolutegravir arm (n=227).

	Efavirenz arm				Dolutegravir arm			
	Univariable associations		Multivariable associations		Univariable associations		Multivariable associations	
Variable(s)	Estimate (95% CI)	p-value	Estimate (95% CI)	p-value	Estimate (95% CI)	p-value	Estimate (95% CI)	p-value
Age (per 5 years increase)	0.075 (-4.249 to 4.399)	0.973	0.873 (-3.554 to 5.300)	0.697	5.000 (1.307 to 8.693)	**0.008**	4.853 (1.389 to 8.317)	**0.006**
Sex								
Female	Referent group				Referent group			
Male	19.349 (3.981 to 34.717)	**0.014**	10.967 (-3.848 to 25.783)	0.146	16.194 (4.882 to 28.710)	**0.006**	8.905 (-1.468 to 19.278)	0.092
Baseline BMI (per 1 kg/m^2^ increase)	-2.009 (-3.085 to -0.933)	**<0.001**	-1.538 (-2.399 to -0.678)	**0.001**	-1.572 (-2.342 to -0.801)	**<0.001**	-1.117 (-1.888 to -0.346)	**0.005**
Baseline CD4 count (per 50 cells/μL increase)	-1.937 (-3.464 to -0.410)	**0.013**	-0.915 (-2.554 to 0.725)	0.272	-1.362 (-2.208 to -0.515)	**0.002**	0.222 (-0.873 to 0.918)	0.961
Baseline HIV-1 RNA (per 1 log_10_ increase)	7.504 (-0.879 to 15.886)	0.079	5.276 (-2.908 to 13.461)	0.205	12.018 (4.950 to 19.085)	**0.001**	8.540 (1.190 to 15.889)	**0.023**
EFV MDI (μg/mL) (per 1 log_10_ increase)	-19.312 (-32.988 to -5.637)	**0.006**	-21.332 (-34.928 to -7.736)	**0.002**	–	–	–	–
DTG AUC_0-24_ (mg·h/L) (per 1 log_10_ increase)	–	–	–	–	-29.976 (-58.814 to -1.137)	**0.042**	-22.793 (-49.810 to 4.223)	0.098

AUC_0-24_ = area under the concentration-time curve, HIV = human immunodeficiency virus, RNA= ribonucleic acid, BMI = body mass index, EFV = efavirenz, MDP = mid-dosing interval plasma concentration, DTG = dolutegravir

## Data Availability

The data that support the findings of this study are available from the corresponding author upon reasonable request.

## What is already known about this subject

- Dolutegravir is associated with more weight gain than efavirenz among antiretroviral therapy (ART) naïve participants in randomised controlled trials.
- It is unclear whether dolutegravir increases weight by off target effects, or if efavirenz toxicity impairs weight gain.

## What this study adds

- We found a strong independent inverse association between efavirenz concentrations and weight gain but there was no independent association between dolutegravir concentrations and weight gain.
- Our findings indicate that weight gain differences between people starting dolutegravir- or efavirenz-based ART are driven by efavirenz toxicity impairing weight gain.
