# Supplementary material for: Concentration-Response Relationships of Dolutegravir and Efavirenz with Weight Change After Starting Antiretroviral Therapy
Source: Br J Clin Pharmacol. Author manuscript; Available in PMC 2022 Mar 1. (PMC7612404; doi:10.1111/bcp.15177)
Supplement: Supplemental material [file EMS140750-supplement-Supplemental_material_.docx]

**Supplemental Material**

**Dolutegravir Population Pharmacokinetic Model**

**Methods**

The pharmacokinetics data were analyzed by non-linear mixed-effects modeling with NONMEM (v7.4.3, ICON Development Solutions, Ellicott City, MD, USA) and first-order conditional estimation with eta-epsilon interaction. Perl-speaks-NONMEM (PSN) (v4.7.0), Piraña (v2.9.7) and R (v3.6.1) were used to aid the modeling process and visualize model diagnostics.

We tested various structural models to describe the pharmacokinetics of dolutegravir including one- and two-compartment disposition models, first-order elimination, and absorption, with or without absorption lag time, and transit compartments. We included between-subject and between-occasion random effects on the model parameters with an assumption of a log-normal distribution.

A combined additive and proportional error model was used to describe the residual errors, with the additive component of the error constrained to be at least 20% of the LLOQ.

To adjust for the effect of body size on the disposition parameters, we added allometric scaling to the model. We tested total body weight and fat-free mass (FFM) with the allometric exponents for clearance and volume parameters fixed to 0.75 and 1 respectively. After the inclusion of allometric scaling, we investigated the following covariate effects on the pharmacokinetic parameters; sex, age, and TAF- vs TDF-based antiretroviral treatment.

Development of the model was guided by the inspection of diagnostic plots, including visual predictive checks (VPC), successful convergence of the estimation algorithm, and a decrease in the objective function value (OFV), which was assumed to follow a chi-square distribution. For an additional degree of freedom (inclusion of one additional parameter), the statistically significant cutoff was an OFV drop of at least 3.84 points, which corresponds to a P of <0.05. We run a non-parametric bootstrap (500 replicates) on the final model to generate the 95% confidence intervals (CI) for the parameter estimates.

We used the developed model to generate post-hoc Bayesian individual steady-state estimates of the area under the concentration-time curve over 24 hours (AUC_0-24_) and unexplained inter individual variability in AUC_0-24_ for each participant with sparse dolutegravir trough concentration data available in the dolutegravir arm. The estimates considered an individual’s pharmacokinetic data and characteristics (i.e., FFM and sex). Individual estimates of AUC_0-24_ were obtained using the formula AUC_0-24_ = Dose/(CL/*F_i_*), where Dose represents the actual dose of dolutegravir given to each individual and CL/*F_i_* represents the individual estimate of oral clearance. For each participant, implausible concentrations were identified based on the absolute value of conditional weighted residual (CWRES) being larger than 4. CWRES follow a normal distribution with mean 0 and variance 1.

**Results**

The population pharmacokinetic model was developed with data from 41 participants with median weight of 68.5 kg (IQR 43 to 121) and age 31 years (IQR 20 to 53). Characteristics for the 41 participants are summarized in Supplemental Table 1.

Dolutegravir exposure was best described by a two-compartment model with first-order elimination and transit compartment absorption. Allometric scaling with fat free mass (FFM) was applied to all clearance and volume parameters with a typical participant described by an FFM of 47 kg.

We estimated a clearance of 0.732 L/h (95% CI 0.666–0.801) with between-subject variability in clearance of 22.3% (14.1–27.8). ﻿The effects of TAF vs TDF on dolutegravir exposure parameters (clearance and bioavailability) were explored, and no statistically significant difference was observed. The goodness of fit plots in Supplemental Figure 3 and the visual predictive check in Supplemental Figure 4 shows that the final model from which we derived AUC_0-24_ adequately described the observed data.

**Supplemental Table 1:** Baseline characteristics of participants in the intensive sampling pharmacokinetic study.

| **Baseline characteristic** | **Median (IQR) or n (%) of volunteers (*n*=41)** |
| --- | --- |
| Age (years) | 31.0 (20.0-53.0) |
| Weight (kg) | 73.8 (49.9-118) |
| Height (cm) | 167 (156-190) |
| Sex, n (%) Male/Female | 27 (66)/ 14 (34) |
| ART regimen |  |
| Dolutegravir/FTC/TDF | 21 (52%) |
| Dolutegravir/FTC/TAF | 20 (49%) |

**Supplemental Table 2:** Comparison of baseline characteristics and percentage weight change from baseline to week 48 between participants in the efavirenz arm enrolled into the pharmacokinetic sub-study versus participants not enrolled (two-sample Wilcoxon rank-sum test for continuous variables and Fisher's exact for categorical variables).

|  | **Participants enrolled from the Efavirenz/FTC/TDF arm**  **(n=158)** | **Participants not enrolled from the Efavirenz/FTC/TDF arm (n=193)** | **p-value** |
| --- | --- | --- | --- |
| **Age (years), median (IQR)** | 32 (28 to 37) | 31 (27 to 37) | 0.462 |
| **Sex (women), n (%)** | 90 (57.0) | 111 (57.5) | 1.000 |
| **Race (black), n (%)** | 158 (100) | 193 (100) | 1.000 |
| **BMI (kg/m^2^), median (IQR)** | 23.8 (20.4 to 27.5) | 22.4 (19.7 to 25.5) | 0.018 |
| **CD4 count (cells/µL), median (IQR)** | 287 (169 to 403) | 313 (185 to 488) | 0.114 |
| **HIV-1 RNA (log_10_), median (IQR)** | 4.4 (3.7 to 5.0) | 4.2 (3.7 to 4.9) | 0.276 |
| **Percentage weight change from baseline to week 48 (%), median (IQR)** | 0.7 (-2.9 to 6.8) | 0.5 (-3.7 to 7.4) | 0.928 |

FTC = emtricitabine , TDF = tenofovir disoproxil fumarate , BMI = body mass index

**Supplemental Table 3:** Comparison of baseline characteristics and percentage weight change from baseline to week 48 between participants in the dolutegravir arm enrolled into the pharmacokinetic sub-study versus participants not enrolled (two-sample Wilcoxon rank-sum test for continuous variables and Fisher's exact for categorical variables).

|  | **Participants enrolled from the Dolutegravir/FTC/TDF arm**  **(n=233)** | **Participants not enrolled from the Dolutegravir/FTC/TDF arm (n=118)** | **p-value** |
| --- | --- | --- | --- |
| **Age (years), median (IQR)** | 32 (27 to 37) | 31 (25 to 36) | 0.120 |
| **Sex (women), n (%)** | 133 (57.1) | 75 (63.6) | 0.253 |
| **Race (black), n (%)** | 233 (100) | 118 (100) | 1.000 |
| **BMI (kg/m^2^), median (IQR)** | 22.9 (20.5 to 27.7) | 22.9 (19.4 to 26.5) | 0.137 |
| **CD4 count (cells/µL), median (IQR)** | 274 (163 to 413) | 276 (169 to 451) | 0.694 |
| **HIV-1 RNA (log_10_), median (IQR)** | 4.4 (3.8 to 4.9) | 4.3 (3.7 to 4.8) | 0.383 |
| **Percentage weight change from baseline to week 48 (%), median (IQR)** | 4.0 (0.6 to 8.1) | 2.3 (-1.2 to 7.4) | 0.221 |

FTC = emtricitabine , TDF = tenofovir disoproxil fumarate , BMI = body mass index

**Supplemental Figure 1:** Goodness-of-fit plots of the final dolutegravir population pharmacokinetic model. Loess smooth curves of the ordinate values are printed in red. a) Observed concentrations vs. population predictions; the line of identity is printed in black. b) Observations vs. individual predictions; the identity line is printed in black. c) Individual weighted residuals (IWRES) vs. individual predictions. d) conditional weighted residuals (CWRES) vs. time post-dose

**
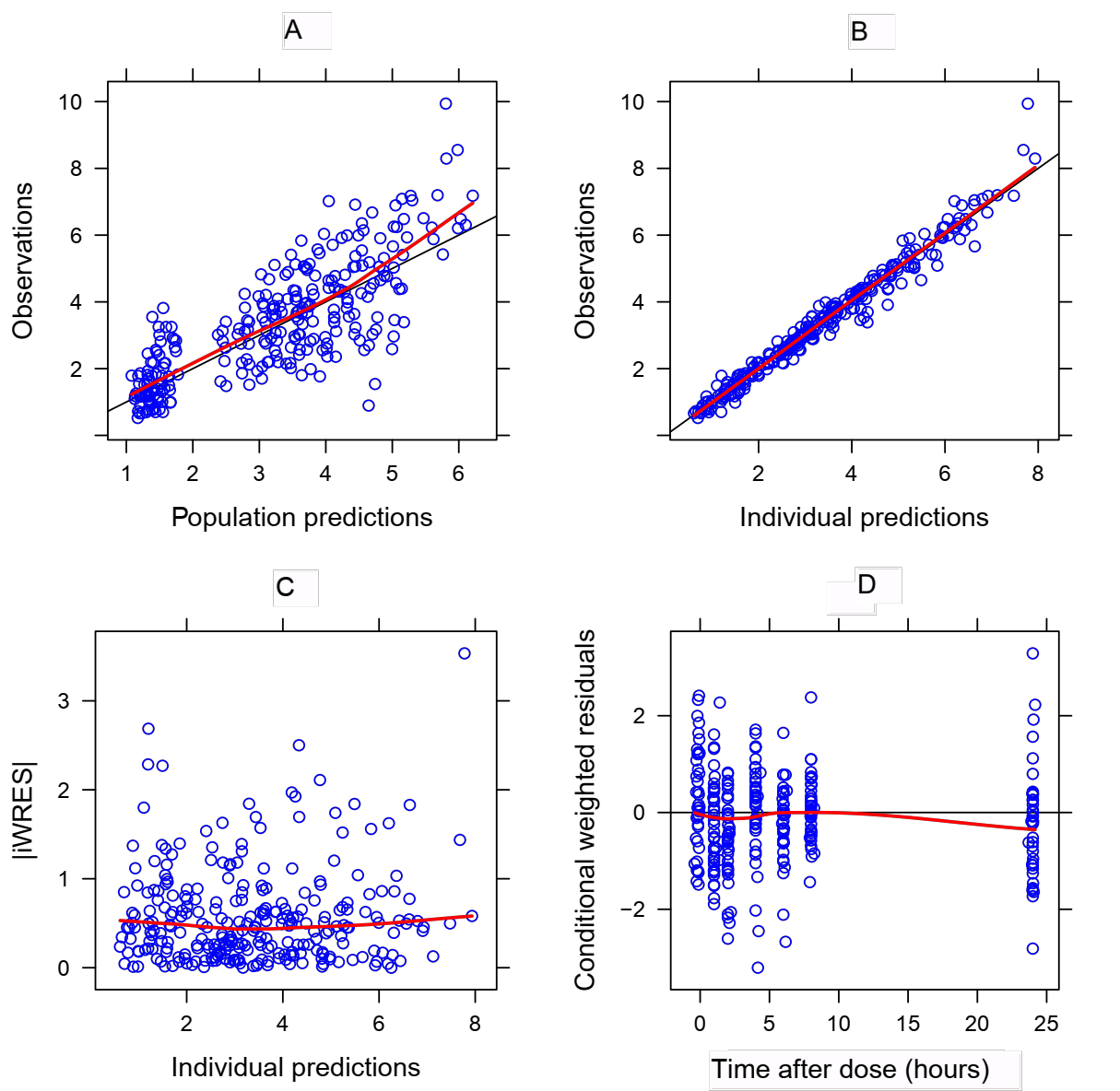
**

**Supplemental Figure 2:** Visual predictive check of the final dolutegravir model applied to all available (sparse and intensively) sampled pharmacokinetic data from the TDF arm. Blue circles represent observed plasma concentrations. The solid line in the middle represents the median observed concentration, the broken lines below and above it represent the 5^th^ and 95^th^ percentiles of the observed concentrations, respectively. The shaded areas around each line represent the 95% confidence interval for the same percentiles based on simulations with the model.

**Supplemental Figure 3:** Participant flow diagram for ADVANCE trial sub-study (FTC = emtricitabine, TDF = tenofovir disoproxil fumarate, LLQ = lower limit of quantification, DXA = Dual energy x-ray absorptiometry, AUC_0-24_ = area under the concentration-time curve, VAT = visceral adipose tissue, SAT = subcutaneous adipose tissue)
